# Vascular abnormalities in fellow eyes of patients with unilateral coats’ disease

**DOI:** 10.1038/s41598-023-45838-1

**Published:** 2023-11-08

**Authors:** Seung Min Lee, Kang Hyun Kim, Hyun Goo Kang, Eun Young Choi, Junwon Lee, Min Kim

**Affiliations:** 1 Department of Ophthalmology, Institute of Vision Research, Gangnam Severance Hospital, Yonsei University College of Medicine, 211 Eonjuro, Gangnam-gu, Seoul, 06273 Republic of Korea; 2 Department of Ophthalmology, Institute of Vision Research, Severance Hospital, Yonsei University College of Medicine, 134 Shinchon-Dong, Seodaemun-gu, Seoul, Republic of Korea

**Keywords:** Eye diseases, Retinal diseases

## Abstract

Coats’ disease is an idiopathic retinal vascular disorder, known to usually occur unilaterally; however, recent studies have highlighted vascular abnormalities in the fellow unaffected eyes. This retrospective study investigated the peripheral vascular features and macular vascular structure of unaffected fellow eyes in patients with unilateral Coats’ disease using multimodal imaging tools. We analysed images of patients, including bilateral ultra-widefield imaging, fluorescein angiography (FA), ultra-widefield FA, or standard fundus photography. Available bilateral optical coherence tomography angiography (OCT-A) images were used for macular vascular structure analysis. OCT-A parameters, including foveal avascular zone (FAZ), perfusion index, and vessel density (VD) in the superficial and deep capillary plexuses (SCP, DCP), were calculated using Image J software. The mean age at diagnosis was 34.5 ± 17.9 years. The mean final best-corrected visual acuity of the affected eyes was logMAR 0.78 ± 0.79, while that of the fellow eyes was logMAR 0.04 ± 0.12. Ten fellow eyes had microaneurysms (47.6%), two had tortuous vessel abnormalities (9.5%), and 11(52.4%) had abnormal vascular findings on FA. Although there was a trend towards larger DCP FAZ (1.201 ± 0.086 vs. 1.072 ± 0.226), and lower DCP VD (8.593 ± 1.583 vs. 10.827 ± 3.392) in the affected eyes as measured by the Cirrus machine, the difference was not statistically significant between affected and fellow eyes when measured using the Zeiss Cirrus machine (*P* = 0.686, *P* = 0.343, respectively). However, when measured with the Spectralis machine, DCP FAZ was larger in affected eyes (0.828 ± 0.426 vs. 0.254 ± 0.092, *P* = 0.002) and DCP VD was lower in affected eyes (6.901 ± 2.634 vs. 17.451 ± 7.207, *P* = 0.002) compared to the fellow eyes, while other parameters showed no significant variations. These findings indicate that there may be subtle vascular abnormalities primarily located in the peripheral regions of the unaffected fellow eyes in patients with unilateral Coats’ disease, while the macular microvasculature remains unaffected.

## Introduction

Coats’ disease, first described by George Coats in 1908, is an idiopathic retinal vascular disorder characterised by vascular telangiectasia and exudative retinopathy^1–4^. It primarily affects young male individuals, but adult-onset cases have also been reported, characterised by milder disease progression than that in childhood-onset cases^5,6^. Typical ophthalmic features of Coats’ disease include retinal telangiectasias, sub- and intraretinal exudates, and exudative retinal detachment^1,6^. Characteristic fundoscopic findings include telangiectatic vessels, aneurysms appearing like light bulbs, peripheral capillary nonperfusion, and peripheral capillary dropout^1^.

The introduction of new imaging modalities with a wider field of view, such as ultra-widefield (UWF) fundus imaging and UWF fluorescein angiography (UWFFA), has allowed more detailed evaluation of retinal vascular structures^7,8^. Optical coherence tomography angiography (OCT-A) is another new noninvasive and less time-consuming tool that allows visualization of the vascular structure of the macula^9–11^. These imaging modalities have been increasingly used for vascular structure analysis in various retinal diseases. Because vascular abnormalities in Coats’ disease often present in the periphery of the retina, these modalities have made detailed evaluation feasible. Consequently, although Coats’ disease is known to usually occur unilaterally^1,2^, recent studies have highlighted vascular abnormalities in the fellow unaffected eyes^12–14^.

Therefore, in this study, we aimed to evaluate the peripheral vascular features and macular vascular structure of the unaffected fellow eyes of patients with unilateral Coats’ disease using multimodal imaging tools.

## Results

Of the 67 screened patients, 63 were diagnosed with unilateral Coats’ disease. Among these, 21 patients with detailed bilateral fluorescein angiography (FA) images were included in the study. Table 1 shows the demographic and clinical characteristics of the study population. The overall mean age at diagnosis was 34.5 ± 17.9 years, and 15 out of 21 patients (71.4%) were of male sex. The mean follow-up duration was 52.5 ± 48.8 (range, 1.8–202.0) months. The disease stage at presentation in the majority of patients was stage 2a (13/21, 61.9%), followed by stage 2b (7/21, 33.3%), and stage 3a1 (1/21, 4.8%). More than half of the patients (11/21, 52.38%) had abnormal vascular findings in the unaffected fellow eyes. The subgroup analysis based on the fellow eye condition is shown in Table 1. There were no statistical differences in the age at diagnosis, sex, laterality, and disease stage between patients with normal and abnormal fellow eye findings (*P* = 0.251, 0.635, 0.670, and 0.361, respectively).

**Table 1:** Demographic and clinical characteristics of the study population.

Baseline characteristics	Overall (n = 21)	Subgroup analysis based on fellow eye condition		
		Fellow eye normal (n = 10)	Fellow eye abnormal (n = 11)	*P* value
Age, yrs, mean ± SD	34.5 ± 17.9 (5–63)	29.4 ± 15.1 (5–50)	39.2 ± 19.7 (14–63)	0.251^a^
Male sex, n (%)	15 (71.4)	8 (80.0)	7 (63.6)	0.635^b^
Coats’ disease laterality Right eye, n (%)	9 (42.9)	5 (50.0)	4 (36.4)	0.670^b^
Disease classification, n (%)				
Stage 2a	13 (61.9)	7 (70.0)	6 (54.5)	0.361^b^
Stage 2b	7 (33.3)	2 (20.0)	5 (45.5)	
Stage 3a1	1 (4.8)	1 (10.0)	–	

Statistical significance was set at *P* < 0.05.

*SD* standard deviation.

^a^Mann–Whitney’s test.

^b^Fisher’s exact test.

**Figure 1:**
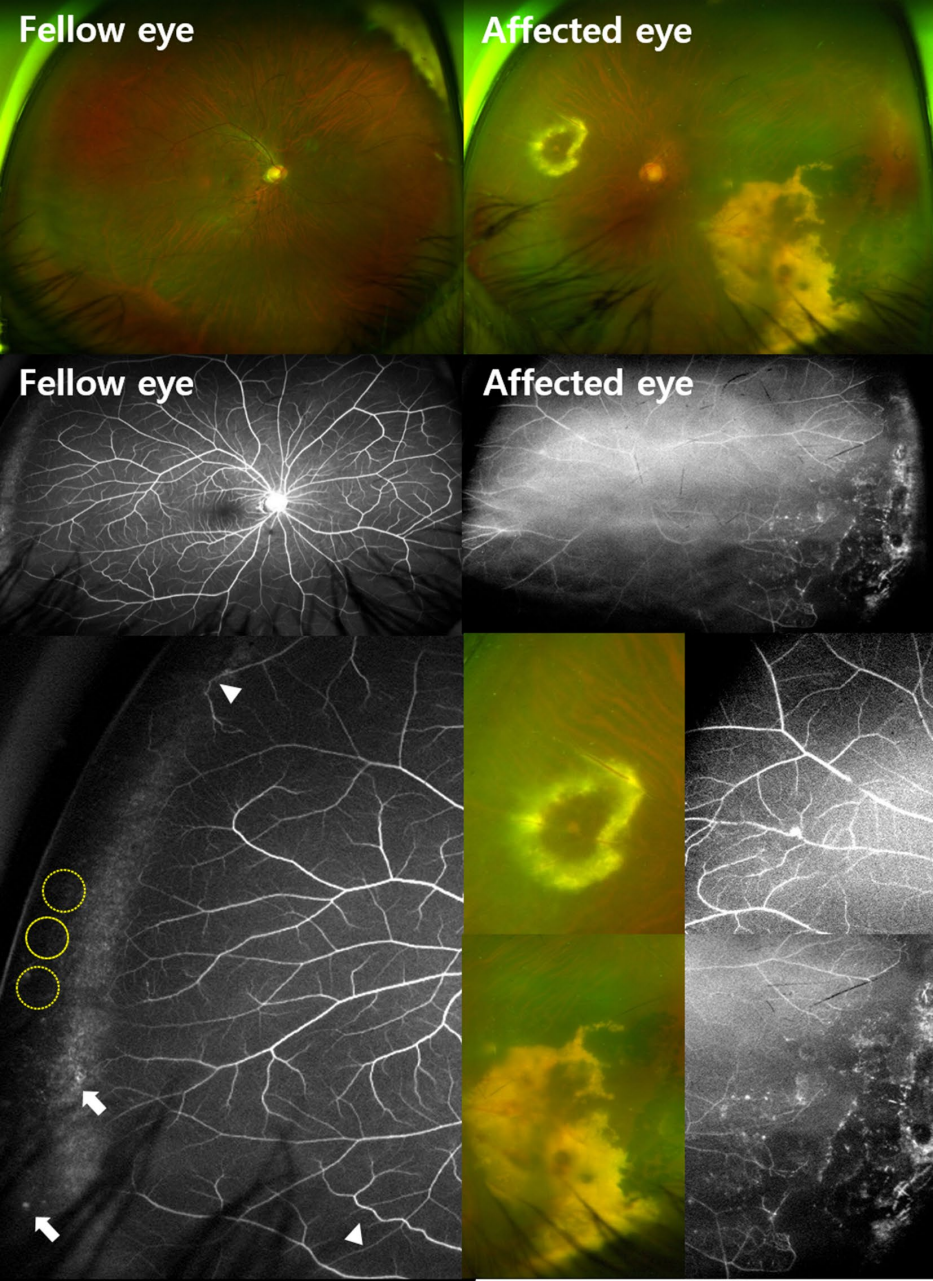
Imaging findings in patient 20. A 58-year old male with Coats’ disease in his left eye (left column). Wide fundus photography of the ‘unaffected’ fellow eye shows no vascular abnormalities, while ultra-widefield image shows microanuerysm (white arrow) and tortuous vascular abnormality (white arrowhead) in the periphery retina. A nonperfusion area exceeding two disc diameters between the temporal vessel termini and the ora serrata is indicated by a yellow dotted circle.

**Figure 2:**
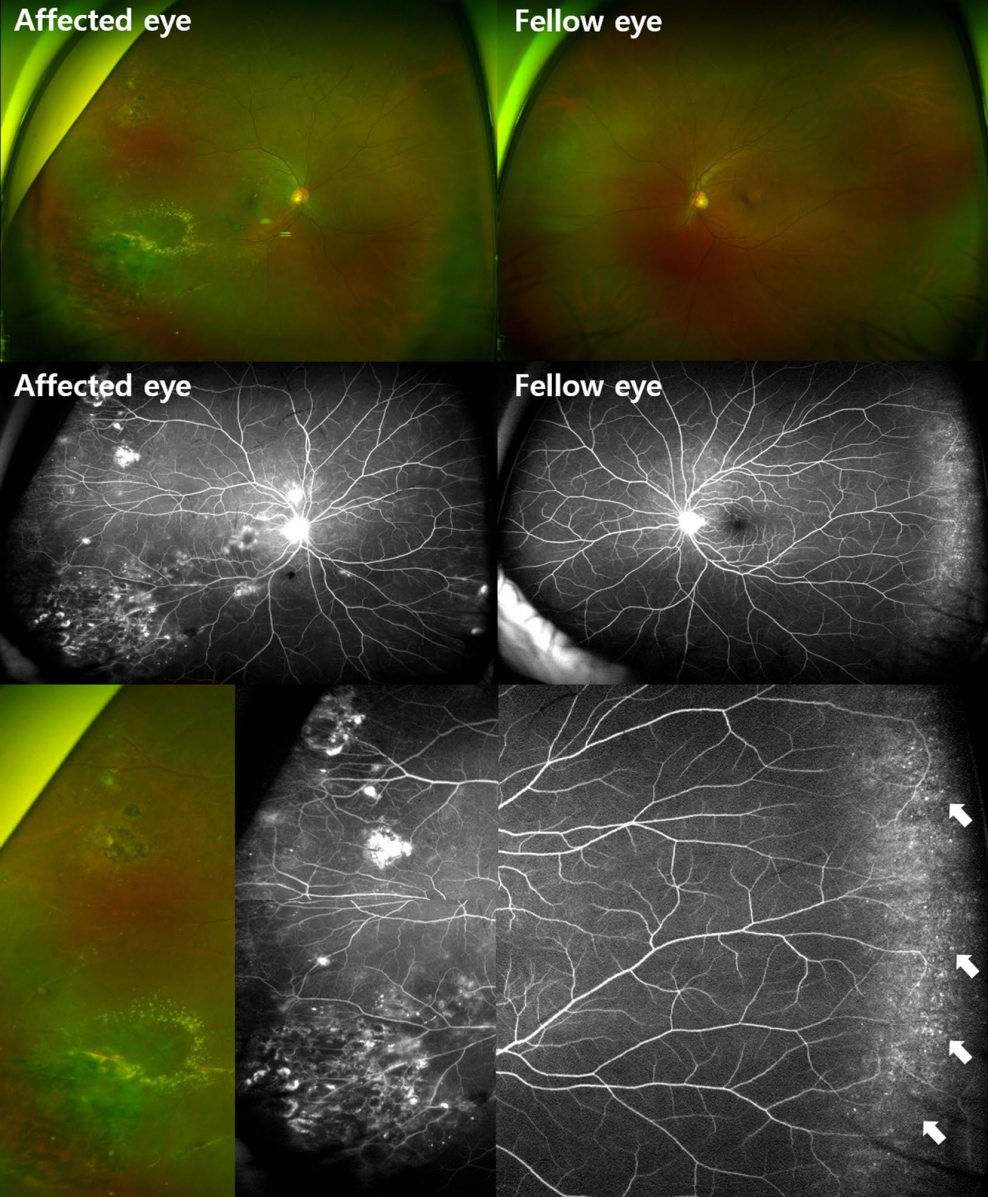
Imaging findings in patient 17. A 56-year old male with Coats’ disease in his right eye (right column). Wide fundus photography of the ‘unaffected’ fellow eye shows no vascular abnormalities, while ultra-widefield image shows multiple microanuerysms in the periphery retina (arrows).

**Figure 3:**
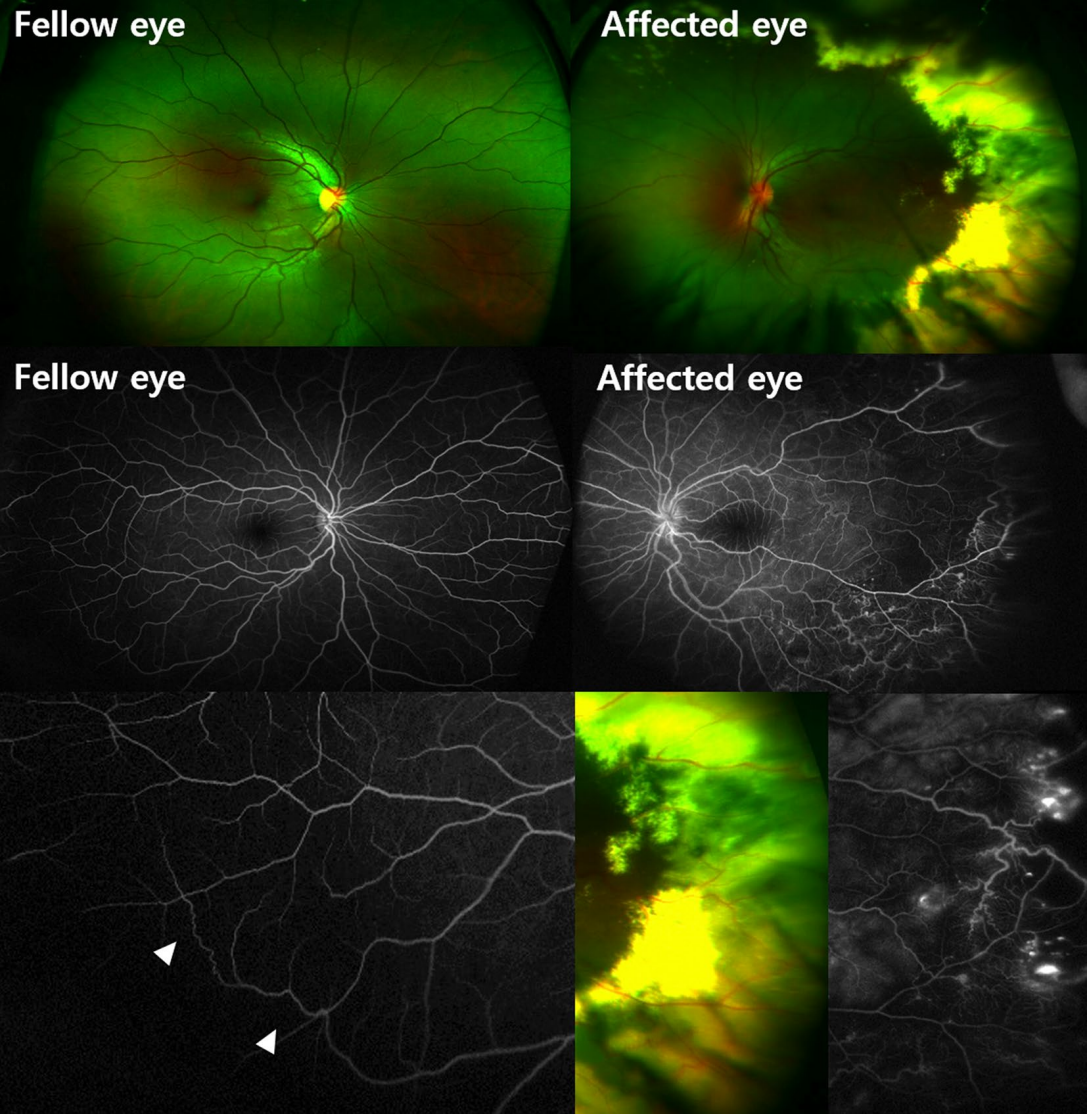
Imaging findings in patient 7. A 18-year old female with Coats’ disease in her left eye (left column). Wide fundus photography of the ‘unaffected’ fellow eye shows no vascular abnormalities, while ultra-widefield image shows multiple tortuous vascular abnormalities in the periphery retina (arrowheads).

Table 2 shows detailed clinical data of the affected eyes in the study population. The most common presenting symptoms/signs in descending order were decreased vision (7/21, 33.3%), floaters (7/21, 33.3%), blurred vision (3/21, 14.3%), routine examination (3/21, 14.3%), and conjunctival injection (1/21, 4.8%). The mean best-corrected visaul acuity (BCVA) of the affected eyes at the final visit was logarithm of the minimum angle of resolution (logMAR) 0.78 ± 0.79 (Snellen 20/125). The bilateral imaging findings in three representative cases are shown in Figs. 1, 2 and 3.

**Table 2:** Clinical data of the affected eyes in patients with unilateral Coats’ disease.

Patient	Age at onset	Affected eye	Stage	Presenting symptoms/signs	Final BCVA, logMAR (Snellen)	Main abnormal vascular findings	Macular edema	Treatment
1	5	OS	2b	Routine examination	3.00 (20/20,000)	Exudation (T), telangiectasia, RH	No	Laser, Cryo
2	6	OS	2a	Decreased vision	0.70 (20/100)	Exudation (T), telangiectasia	No	Laser, Cryo
3	12	OS	2a	Decreased vision	0.10 (20/25)	Exudation (IT), telangiectasia, RH	No	Laser, Cryo
4	14	OS	2b	Decreased vision	0.30 (20/40)	Exudation (N), telangiectasia, RH	Yes	Laser, Anti-VEGF
5	14	OS	2b	Decreased vision	2.00 (20/2000)	Exudation (M, T, N), telangiectasia, RH	Yes	Laser, Cryo, PPV
6	14	OD	2a	Decreased vision	0.22 (20/33)	Exudation (I), telangiectasia	No	Observation
7	17	OS	2a	Floaters	1.40 (20/500)	Exudation (T, S, I), telangiectasia, RH	Yes	Laser, Cryo, Anti-VEGF, PSTI
8	20	OS	2a	Floaters	0.82 (20/133)	Exudation (N), telangiectasia, RH	Yes	Laser, Anti-VEGF
9	27	OD	2b	Vision blurring	1.40 (20/500)	Exudation (N, S, M), telangiectasia, RH	Yes	Laser, Anti-VEGF
10	31	OD	2a	Floaters	0.10 (20/25)	Exudation (T), telangiectasia, VH, RH	No	Laser
11	32	OD	2a	Routine examination	0.00 (20/20)	Exudation (T), telangiectasia, RH	No	Laser, Anti-VEGF
12	35	OD	2a	Vision blurring	0.00 (20/20)	Exudation (T), telangiectasia	No	Laser, Anti-VEGF, PSTI
13	41	OS	3a1	Vision blurring	0.70 (20/100)	Exudation (S), telangiectasia, RH	No	Laser, Anti-VEGF
14	42	OS	2a	Floaters	0.40 (20/50)	Exudation (T), telangiectasia	No	Laser
15	42	OD	2a	Decreased vision	0.22 (20/33)	Exudation (T), telangiectasia, RH	No	Laser, Anti-VEGF, PSTI
16	47	OD	2b	Routine examination	1.70 (20/1000)	Exudation (T), telangiectasia	Yes	Laser, Anti-VEGF, PSTI
17	47	OD	2a	Floaters	0.15 (20/29)	Exudation (T), telangiectasia, RH	No	Laser, Anti-VEGF
18	51	OS	2b	Conjunctival injection	1.40 (20/500)	Exudation (ST), telangiectasia, RH	Yes	Laser
19	56	OD	2a	Floaters	0.05 (20/22)	Exudation (S), telangiectasia	No	Laser
20	57	OS	2a	Decreased vision	0.70 (20/100)	Exudation (IT, N), telangiectasia, RH	No	Laser, Anti-VEGF
21	63	OS	2b	Floaters	1.10 (20/250)	Exudation (S, T), telangiectasia, RH	Yes	Laser, Anti-VEGF

*BCVA* best-corrected visual acuity, *logMAR* logarithm of the minimum angle of resolution, *OS* left eye, *OD* right eye, *VH* vitreous haemorrhage, *RH* retinal haemorrhage, *VEGF* vascular endothelial growth factor, *Cryo* cryotherapy, *PPV* pars plana vitrectomy, *PSTI* posterior subtenon injection.

The quadrant location of exudation is indicated in the parenthesis: *T* temporal, *IT* inferotemporal, *I* inferior, *N* nasal, *S* superior, *ST* superotemporal, *M* macula.

The clinical data of the unaffected fellow eyes of our patients are shown in Table 3. The mean BCVA of the fellow eyes at the final visit was logMAR 0.04 ± 0.12 (Snellen 20/20). Ten fellow eyes had microaneurysms (47.6%) (Figs. 1 and 2), two had tortuous vessel abnormalities (9.5%) (Figs. 1 and 3), and 11 (52.38%) had abnormal vascular findings on FA. However, none of the fellow eyes showed leakage on FA or macular edema. Only one patient (Patient 20) had images capturing the far periphery, including the ora serrata. The images revealed a nonperfusion area exceeding two disc diameters between the temporal vessel termini and the ora serrata, as highlighted in Fig. 1. Within a cohort of 21 patients, 7 manifested as childhood-onset cases with diagnoses established prior to reaching the age of 18, while the remaining 14 were classified as adult-onset cases. The occurrence of fellow eye vascular abnormalities among pediatric patients amounted to 4 out of 7, accounting for 57.1%. Similarly, adult patients who displayed fellow eye vascular abnormalities constituted 7 out of 14, equating to 50%.

**Table 3:** Clinical data of the unaffected fellow eyes in patients with unilateral Coats’ disease.

Patient	Final BCVA, logMAR (Snellen)	Main abnormal vascular findings	Leakage on FA	Macular edema
1	0.10 (20/25)	None	No	No
2	0.00 (20/20)	Microaneurysms	No	No
3	0.00 (20/20)	None	No	No
4	0.00 (20/20)	Microaneurysms	No	No
5	0.00 (20/20)	Microaneurysms	No	N/A
6	0.00 (20/20)	None	No	No
7	0.10 (20/25)	Tortuous abnormalities	No	No
8	0.00 (20/20)	None	No	No
9	0.00 (20/20)	Microaneurysms	No	No
10	0.10 (20/25)	None	No	No
11	0.00 (20/20)	None	No	No
12	0.00 (20/20)	Microaneurysms	No	No
13	0.52 (20/67)	None	No	No
14	0.00 (20/20)	None	No	No
15	0.00 (20/20)	None	No	No
16	0.00 (20/20)	None	No	No
17	0.00 (20/20)	Microaneurysms	No	No
18	0.00 (20/20)	Microaneurysms	No	No
19	0.00 (20/20)	Microaneurysms	No	No
20	0.00 (20/20)	Microaneurysms, Tortuous abnormalities	No	No
21	0.00 (20/20)	Microaneurysms	No	No

*BCVA* best-corrected visual acuity, *logMAR* logarithm of the minimum angle of resolution, *FA* fluorescein angiography, *N/A* not available.

In the cases of patients 7, 9, 13, 18, and 19, follow-up FA images were obtained with a time difference of at least one year from the initial FA examination (at least one year up to 5 years). These subsequent images did not reveal any changes confirming disease progression or a diagnosis of Coats' disease in the fellow eye, except for subtle vascular abnormalities that were still observed from the initial presentation. Furthermore, there were no notable differences in the progression of fellow eye presentations based on variations in the age of onset. As for patients 6 and 15, whose fellow eyes did not exhibit any vascular abnormalities at presentation, follow-up FA images obtained 3 years later still showed a normal fundus in the fellow eye.

Bilateral OCT-A images were available for 10 of the 21 included patients. All 10 included patients had disease stages between 2a and 2b. Among 10 patients, six were evaluated with Spectralis HRA-OCT-A, and the rest four were evaluated with Zeiss Cirrus 5000 HD-OCT. Table 4 shows the OCT-A parameters used for macular vascular evaluation in the affected and unaffected fellow eyes. Although there was a trend towards larger deep capillary plexus (DCP) foveal avascular zone (FAZ) and lower DCP vessel density (VD) in the affected eyes, the difference was not statistically significant between affected and fellow eyes when measured using the Zeiss Cirrus machine. When measured with the Spectralis machine, DCP FAZ was larger in the affected eyes (0.828 ± 0.426 vs. 0.254 ± 0.092, *P* = 0.002), and DCP VD was lower in the affected eyes (6.901 ± 2.634 vs. 17.451 ± 7.207, *P* = 0.002) compared to the fellow eyes, while other parameters showed no significant variations.

**Table 4:** Optical coherence tomography angiography analysis.

Zeiss Cirrus 5000 HD-OCT			
	Affected eye FAZ (n = 4)	Fellow eye FAZ (n = 4)	*P* value
SCP FAZ (mm^2^)	0.351 ± 0.155	0.309 ± 0.081	0.886
DCP FAZ (mm^2^)	1.201 ± 0.086	1.072 ± 0.226	0.686
SCP Perfusion index	0.316 ± 0.054	0.342 ± 0.061	0.486
DCP Perfusion index	0.401 ± 0.374	0.390 ± 0.345	1.000
SCP Vessel density (mm^−1^)	12.820 ± 3.835	15.090 ± 4.246	0.486
DCP Vessel density (mm^−1^)	8.593 ± 1.583	10.827 ± 3.392	0.343

Spectralis HRA + OCT-A			
	Affected eye FAZ (n = 6)	Fellow eye FAZ (n = 6)	*P* value
SCP FAZ (mm^2^)	0.201 ± 0.115	0.225 ± 0.084	0.937
DCP FAZ (mm^2^)	0.828 ± 0.426	0.254 ± 0.092	**0.002**
SCP Perfusion index	0.393 ± 0.185	0.329 ± 0.058	0.699
DCP Perfusion index	0.893 ± 0.020	0.808 ± 0.095	0.065
SCP Vessel density (mm^−1^)	20.758 ± 15.464	18.633 ± 8.355	0.818
DCP Vessel density (mm^−1^)	6.901 ± 2.634	17.451 ± 7.207	**0.002**

Data are presented as mean ± standard deviation.

*SCP* superficial capillary plexus, *FAZ* foveal avascular zone, *DCP* deep capillary plexus.

Statistical significance was set at *P* < 0.05 (Mann–Whitney’s test).

Significant values are in [bold].

## Discussion

In this study, more than half of the patients with unilateral Coats’ disease (11/21, 52.38%) had peripheral vascular abnormalities in the unaffected fellow eyes. However, there were no central retinal vascular abnormalities; thus, the visual acuity in the fellow eyes was preserved (logMAR 0.04 ± 0.12, Snellen 20/20).

In their study of the peripheral vascular morphology of the normal retina using UWFFA, Seo et al.^15^ found that 30–40% of subjects with both normal eyes had bilateral microvascular abnormalities, such as telangiectasia, microaneurysms, and vascular leakage. In addition, normal eyes of subjects with contralateral eyes affected by vascular disease showed a relatively higher degree of microvascular abnormalities (40–60%). In our study, the proportion of fellow normal eyes with peripheral vascular abnormalities was 52.4%, which is higher than that for bilateral normal eyes and similar to that for unilateral normal eyes in subjects with vascular disease in contralateral eyes reported by Seo et al.^15^ The percentage of childhood-onset cases and adult-onset cases with fellow eye vascular abnormalities separately exceeded 50%, indicating a higher likelihood of abnormalities. Notably, the percentages of abnormalities in both the child and adult groups fell within the range of 40–60%. Brockmann et al. investigated the peripheral vascular abnormalities in unilateral Coats’ disease using UWFFA and found that all normal fellow eyes had vascular abnormalities^14^. Our results did not indicate such strong association between unilateral Coats’ disease and vascular abnormalities in the fellow eyes. Furthermore, during the follow-up period, none of the fellow eyes developed observable Coats’ disease, and the subtle vascular abnormalities did not appear to progress further.

Several studies have demonstrated foveal microvascular alterations in normal eyes and those with Coats’ disease using OCT-A. Stanga et al.^13^ compared the OCT-A parameters of 13 fellow eyes of patients with unilateral Coats’ disease with those of 10 normal controls and concluded that fellow eyes in unilateral Coats’ disease had larger SCP FAZs than controls. Brockmann et al.^14^ found that the mean FAZ of 19 fellow eyes was 0.20 mm^2^, which was below the normal range (0.24–0.35 mm^2^)^16,17^. Daruich et al.^18^ found larger FAZ and decreased VD in affected eyes compared with fellow and control eyes. Similar to Daurich et al. we identified a significant difference exclusively in the measurement of DCP FAZ and DCP VD using the Spectralis machine (*P* = 0.002 for both). The affected eye displayed a larger DCP FAZ and a lower DCP VD in comparison to the fellow eye. Daruich et al. posited that the unilateral nature of the disease is evident, given that only the affected eye exhibits an enlarged FAZ and reduced VD^18^. Nonetheless, our study showed subtle vascular abnormalities, primarily confined to the peripheral retina in the unaffected fellow eyes with negligible progression over years.

Although the DCP FAZ was larger in the affected eyes (1.201 ± 0.086 vs. 1.072 ± 0.226), and the DCP VD was lower in the affected eyes (8.593 ± 1.583 vs. 10.827 ± 3.392) as measured by the Cirrus machine, there was no statistically significant difference in these measurements between affected and fellow eyes (*P* = 0.686, *P* = 0.343, respectively). The lack of a statistically significant trend could be linked to the limited sample size in the Cirrus OCTA evaluation. Further research is needed to enhance our understanding of macular vasculature using the Cirrus OCTA machine.

This study has several limitations. First, due to the retrospective design, selection bias might have been introduced. Second, the number of patients was relatively small, which limits the generalizability of the findings. Third, two different imaging devices were used for FA and OCT-A. Therefore, future prospective multicenter studies with a larger sample size are required to confirm our findings.

In patients with unilateral Coats’ disease, subtle vascular abnormalities might be present in the unaffected fellow eyes, suggesting potential bilateral involvement. These abnormalities are primarily confined to the retinal periphery and do not appear to progress rapidly, nor do they significantly affect macular vascular structure in the unaffected fellow eyes regardless of onset age, but their presence should be acknowledged by physicians.

## Methods

This retrospective study was conducted based on the clinical records of patients from two referral-based hospitals, Severance Eye Hospital and Gangnam Severance Hospital, both affiliated with the Yonsei University College of Medicine in Seoul, Korea. The study adhered to the tenets of the Declaration of Helsinki, and was approved by the Gangnam Severance Hospital Institutional Review Board (No. 3-2020-0139). The need for informed consent was waived owing to the retrospective design of the study.

Patients diagnosed with Coats’ disease who were followed up between July 2000 and April 2020 were screened. For each screened case, we reviewed the complete electronic medical records and multimodal imaging data to confirm the diagnosis. Patients with unilateral Coats’ disease and available bilateral UWF fundus images (Optos; Optos Plc, Dunfermline, Scotland, UK), FA or UWFFA images (Optos Silverstone; Optos Plc), or standard fundus images (Spectralis; Heidelberg Engineering, Heidelberg, Germany) were included. FA images captured by a standard fundus camera with a 30–50° field of view were included if sufficient peripheral vascular structures in both eyes were visualised. Patients with inadequate/unavailable data or images or ocular pathologies in the fellow eye were excluded.

The following data were analysed: demographics, age at diagnosis, disease stage at presentation based on the classification proposed by Shields et al.^2^ follow-up duration, BCVA at the initial and final stages of Coats’ disease, symptoms, fundus examination findings, multimodal fundus imaging such as FA, OCT, and OCT-A scans, and treatment modalities.

Macular imaging was performed using two different devices: Spectralis HRA + OCT-A (4.4 × 2.9 mm scans; Heidelberg Engineering) and Zeiss Cirrus 5000 HD-OCT (3 × 3 mm scans; Zeiss Meditec. Inc., Jena, Germany). Patients with complete angiographic images of both eyes were included in a subanalysis to evaluate the changes in the macular microvasculature. The following OCT-A parameters were analysed on both types of scans (The Spectralis scanned images were cropped to 1.5 × 1.5 mm for analysis, and the Cirrus scanned images were used without cropping): FAZ, perfusion index (PI), and VD of the SCP and DCP. The SCP and DCP FAZs were measured after delineating the borders of vessel-free central part of the macula in both SCP and DCP manually using Image J software.

The PI was automatically calculated using image binarization, and VD (mm^−1^) was calculated after skeletonization of the binarized image according to previously described methods^19^.

Statistical analysis was performed using IBM SPSS Statistics (version 25.0; IBM Corp., Armonk, NY, U.S.A.). Mann–Whitney’s and Fisher’s exact tests were used for all comparisons. Where appropriate, 95% confidence intervals were also calculated. Statistical significance was set at *P* < 0.05.

### Institutional review board statement

The study was conducted in accordance with the Declaration of Helsinki, and approved by the Institutional Review Board of the Gangnam Severance Hospital (IRB approval number: No. 3-2020-0139).

### Informed consent

Patient consent was waived due to the retrospective manner of this study by the Gangnam Severance Hospital Institutional Review Board (IRB approval number: No. 3-2020-0139).

## Data Availability

The datasets generated during and/or analysed during the current study are available from the corresponding author on reasonable request.
